# Travelling-Wave Dipolophoresis: Levitation and Electrorotation of Janus Nanoparticles

**DOI:** 10.3390/mi12020114

**Published:** 2021-01-22

**Authors:** Touvia Miloh, Jacob Nagler

**Affiliations:** School of Mechanical Engineering, University of Tel-Aviv, Tel-Aviv 69978, Israel; syankitx@gmail.com

**Keywords:** electrokinetics of metallodielectric Janus particles, dipolophoresis, dielectrophoresis, induced-charge electrophoresis, travelling waves and non-uniform fields, electrorotation, levitation and stability

## Abstract

We present a theoretical study of the hydrodynamic and electrokinetic response of both metallic spherical polarized colloids as well as metallodielectic Janus particles, which are subjected to an arbitrary non-uniform ambient electric field (DC or AC forcing). The analysis is based on employing the linearized ‘standard’ model (Poisson–Nernst–Planck formulation) and on the assumptions of a ‘weak’ field and small Debye scale. In particular, we consider cases of linear and helical time-harmonic travelling-wave excitations and provide explicit expressions for the resulting dielectrophoretic and induced-charge electrophoretic forces and moments, exerted on freely suspended particles. The new analytic expressions thus derived for the linear and angular velocities of the initially uncharged polarizable particle are compared against some available solutions. We also analyze the levitation problem (including stability) of metallic and Janus particles placed in a cylindrical (insulating or conducting) pore near a powered electrode.

## 1. Introduction

One of the preferred techniques for manipulating and controlling phoretic motions of micro/nano polarizable particles freely suspended in an electrolyte, is by means of externally applying ambient electric fields. The applied field can be steady (DC) or time-harmonic (AC), as well as uniform (constant) or spatially non-uniform. The nanoparticle (NP) itself can be conducting or dielectric, uncharged or initially charged, chemically active or inert, homogeneous or non-homogeneous (i.e., meta-material). In this study, we theoretically consider the case of a metallic (perfectly conducting) spherical NP, as well as metallodielectric (MD) Janus particle (JP) comprising of two hemispheres, one metallic and the other dielectric. Both NP and JP are subjected to an arbitrary time-harmonic non-uniform electric field and are assumed inert and initially uncharged. Nano-fluidic applications of such colloids are abundant, especially those involving JP due to their inherent symmetry-breaking features and potential use as cargo carriers (see for example the recent reviews [1,2]). Nevertheless, it should be mentioned that in spite of the current interest in JP electrokinetics and the growing number of publications on the subject, theoretical studies on the dynamics (i.e., induced linear and angular velocities) of JP’s under general non-uniform AC excitations, are rather scarce [3].

When exposed to a non-uniform ambient field, an initially uncharged polarized colloid experiences a dielectrophoretic (DEP) force and torque, determined by the multipole system (including the leading dipole term) within the NP and the corresponding partial derivatives of the ambient potential evaluated at these singularities [4]. Due to polarization, a distribution of induced charges (Poisson equation) is created in the electrolyte (decaying exponentially away from the NP) by the electric field. The above induced-charge density interacts with the electric field and inflicts a low-Reynolds (creeping) fluid motion around the NP. The resulting force (integration of viscous stresses over the NP) is defined as the ‘induced –charge electrophoretic’ force (ICEP) acting on the colloid [5,6]. The sum of these two forces (DEP +ICEP) has been coined by [7] as the dipolophoretic (DIP) force. At least for spherical polarized colloids (especially at low forcing frequencies), DEP and ICEP generally act in opposite directions. Interestingly, it has been shown by [8], that for a *constant-gradient* field (i.e., linear in Cartesian coordinates), DEP and ICEP for a spherical NP precisely cancel each other so that the total DIP is practically null! However, as demonstrated in [9,10], this result holds only under the limit of infinitely thin electric double layer (EDL) and for time-independent (DC) electric forcing.

In most cases, the applied fields are taken as uniform. They may be unidirectional or have two out of phase uniform components, acting along orthogonal directions resulting in electrorotation (ROT) [4,11]. In these cases, the DEP force is null but there is a finite DEP torque leading to particle rotation. Explicit expressions for the DIP angular velocity of, say metallic nanowire [12], sphere [13,14,15] or tri-axial ellipsoid [16], can be found by separately considering the DEP and ICEP contributions to the torque exerted on the NP. Alternately, the ambient field may combine uniform and ‘constant-gradient’ terms [8,9,17] (typical for active colloids [18]), where both DEP and ICEP act simultaneously in opposite directions. The most general electric forcing however, involves arbitrary time-harmonic (single or multiple frequencies) non-uniform ambient fields, acting on a colloid of unrestricted EDL. The underlying nonlinear electrostatic problem is first solved by linearization and employing the PNP standard model based on the ‘weak’ field assumption [19]. The hydrodynamic problem is next analyzed by considering the non-homogeneous Stokes equation with a Columbic forcing.

The special case of an infinitely polarizable (metallic) free spherical NP placed under AC excitations [5,19,20], is of particular interest since it is amenable to analysis and so is (for the same reason) the special case of a travelling- wave electric forcing [4,11]. One of the earlier studies on sinusoidal time-harmonic travelling-wave electrokinetics, is due to Huang et al. [21], coining the ‘twDEP’ terminology and demonstrating that unlike conventional DEP, twDEP is related to the imaginary rather to the real part of the NP dipole- term. The idea of pumping fluids by means of travelling-wave electro-osmosis, using an array of equally spaced planar or spiral elec-trodes, was proposed in several studies [22,23,24,25]. The corresponding twDIP axisymmetric problem of freely suspended perfectly conducting spherical NP, placed along the center of a cylindrical pore, was analyzed by Miloh and Boymelgreen [26]. Recently, a theoretical/numerical twDIP study of free metallic spherical (or cylindrical) NP’s, lying in unbounded space and subject to a sinusoidal travelling wave AC electric forcing, has been also presented by Flores et al. [27]. However, the above mentioned twDIP studies, can be considered only as ‘long-wave length’ approximations of the full problem (by considering only the first two linear terms) and practically fail when the typical wavelength of the electric forcing, is of the same order as the size of the NP (Rayleigh’s assumption). One of the main results of the present study, is obtaining a closed-form (Bessel function) results for the twDIP force and torque acting on a metallic spherical NP under a general 3D linear or helical travelling-wave electric forcing. These new results are exact in the sense that they are valid for arbitrary wavelengths and are shown to reduce (to leading-order) to the available approximate (long-o wave) solutions for metallic spherical NP’s.

Unlike homogeneous NP’s, DIP theoretical studies on non-homogeneous (two-face) Janus particles are scarce [28,29], due to assitional symmetry breaking complexities. Indeed, most of the recent publications (mostly experimental) on JP’s electrokinetics, consider metallodielectic (including effect of coating) spherical particles under a *uniform* DC ac AC fields [3,8,18,30,31,32,33,34,35,36,37,38]. As stated, the growing interest in JP’s dynamics is connected with their special self-propulsion features as cargo carriers, mobile electrodes and unique frequency dispersion characteristics. A spherical JP that is subjected to a uniform field will not experience a DEP force (ignoring boundary or wall effects) and any non-symmetric particle (contrary to a *spherical* NP), will acquire a finite phoretic velocity due to ICEP, as first demonstrated in the DC limit by Squires and Bazant [8]. When exposed to a non-uniform ambient field (such as linear or helical travelling wave excitations), the JP is subjected to both DEP and ICEP resulting in an induced phoretic motion ( translation or rotation). Nevertheless, as demonstrated in the sequel, the DIP response of a JP is essentially different from that of an homogeneous NP, even under the same electric forcing. By taking advantage of the large disparity between the dielectric properties of the two hemispheres comprising the metallodielectric JP, we are able to further simplify the DIP analysis [28,29] and present for the first time an explicit DIP solution for a spherical JP excited by an arbitrary travelling wave. We also consider the pertinent DEP levitation problem [4,39,40,41,42,43] of a freely suspended JP placed axi-symmetrically in a cylindrical pore near a powered circular electrode. In addition to the practical problem of electric levitation, there is much interest in other modes of physical levitation, such as acoustic and optical techniques [44,45,46] for manipulating free nanoparticles under AC fields in a gaseous environment. By analyzing the stability (radial and axial) of the above DEP levitation problem, we demonstrate that a spherical JP is more responsive to levitation, since its height is 2–3 larger compared to that of an equivalent NP (same forcing). We also provide a simple expression for this equilibrium elevation in terms of the physical parameters.

In this work we present a theoretical framework for determining the dynamic response (forces and torques) of spherical metallic NP’’s or metallodielectric Janus particles, freely suspended in an unbounded electrolyte that are exposed to a spatially non-homogeneous time- harmonic ambient electric field. Analytic expressions are provided for both the DEP and ICEP dynamic loads (forces and torques) acting on a single colloid, thus providing the total DIP phoretic response of the NP (i.e., linear and angular velocities), under the creeping flow (Stokes) hypothesis. In particular, we consider cases of NP or JP that are subjected to linear or helical travelling wave AC excitations and provide exact solutions for the loads in terms of the forcing wavelength and frequency. The different dynamic response between NP and JP to the same electric excitations, are also discussed in the context of the levitation problem of an isolated colloid placed in a vertical cylindrical pore above a powered electrode. Explicit expressions are provided for the equilibrium levitation height and passive stability in both radial and axial directions.

The structure of the paper is as follows: In Section 2, we provide a summary of the general methodology for solving the non-linear electrostatic problem of an initially uncharged freely suspended spherical NP or JP lying in an unbounded symmetric binary electrolyte. Linearization is then enforced by assuming ‘weak’ field AC excitations (‘standard’ model), resulting in a Robin-type boundary condition applied on the surface of the polarizable colloid. The DEP problem is first discussed in Section 3, by obtaining explicit expressions for the internal higher -order multipoles (DC and AC) in axisymmetric and non-symmetric electric excitations. In particular we demonstrate the above methodology for linear and helical travelling- wave forcing. Next, we consider in Section 4, the corresponding ICEP problem for the same electric forcing using Teubner’s [47] approach under the assumption of an infinitely thin EDL. The frequent case of the DIP torque exerted on NP or JP under helical (circumferential) wave forcing, is presented in Section 5 and compared against the available electrorotation (ROT) solutions. The special shape of a metallodielectric JP, consisting of two hemispheres which have unique symmetry-breaking properties affecting both DEP and ICEP, is discussed in Section 6 including a comparison to the known DC- ICEP solution under a uniform field forcing. Finally, we present, in Section 7, detailed stability analysis of the pertinent levitation problem of both NP and JP placed symmetrically in a vertical cylindrical pore (insulating or conducting walls) above a powered circular electrode. It is demonstated that JP’s are more responsive to levitation compared to NP’s (by a factor 2–3) under the same forcing. We conclude with a short Section 8, including a summary and discussion.

## 2. General Formulation

We consider a spherical metallodielectric (MD) micro/nano Janus particle (JP), which is exposed to an ambient arbitrary non-uniform alternative current (AC) electric field (such as a travelling- wave). The free JP of radius *a* consists of two hemispheres with large contrast between the corresponding permittivities and is suspended in a symmetric monovalent aqueous unbounded electrolyte. Using a spherical coordinate system (R,θ,φ) centered at the JP, the general imposed non-homogenous harmonic electric field can be conveniently expressed as:

(1) $${\bar{\chi }}_{am}\left(R,\theta ,\varphi \right)=-\overset{\infty }{\underset{n=1}{\sum }}\overset{2n+1}{\underset{m=0}{\sum }}A_{n}^{m}{\tilde{R}}^{n}P_{n}^{m}\left(\mu \right)e^{im\varphi };\;\;\tilde{R}=R/a$$

where $A_{n}^{m}$ are complex amplitudes, μ=cosθ and $P_{n}^{m}\left(\mu \right)$ denote the associate Legendre polynomials. Written in terms of a Cartesian coordinate system, having the same origin, $x_{1}=R\mu$ and $x_{2}+ix_{3}=R\sin\theta e^{i\varphi }$, the AC electric potential χ of frequency ω can be expressed in terms of its phasor Equation (1), as χ=Re{χ¯e−iωt}.

The partially polarized initially uncharged JP is assumed impervious to both anions and cations. Because of polarization and by virtue of Gauss’s law, an induced-charge distribution [5,6] will be formed around the particle, exhibiting an exponential decay away from the polarized JP. The induced-charge density *Q* is governed by the Helmholtz equation and depends on the Debye scale $\lambda_{0}$, which for most electrolyte solutions is of the order of few tenths of a nanometer. By ignoring surface conductance (small Dukhin number) and under the assumptions of a ‘weak’ field (i.e., below the thermal scale) and thin ($\lambda_{0}\ll a$) EDL (electric double layer), which corresponds to the traditional ‘Standard model’ [6,7,8,9,10] can be written in terms of its phasor as:

(2) $$2\bar{\phi }=-{\left(\frac{\lambda }{\lambda_{0}}\right)}^{2}\bar{Q}+\bar{\chi };\;\frac{1}{\lambda^{2}}=\frac{1}{\lambda_{0}{}^{2}}-\frac{i\omega }{D}$$

where $\left(\bar{Q},\bar{\phi },\bar{\chi }\right)$ satisfy $\lambda^{2}\nabla^{2}\bar{Q}=\bar{Q}=-2\lambda_{0}^{2}\nabla^{2}\bar{\phi }$ and $\nabla^{2}\bar{\chi }=0$. Here λ denotes the complex Debye scale and D represents the diffusivity of the symmetric electrolyte. Thus, following [10], $\bar{Q}$ (Helmholtz) and $\bar{\chi }$ (Laplace) are expressed as:

(3) $$\begin{array}{l} \bar{Q}=-2\sum_{n=1}^{}\sum_{m=0}^{}C_{n}^{m}{Κ}_{n}\left(\tilde{R}/\lambda \right)P_{n}^{m}\left(\mu \right)e^{im\varphi };\\ \bar{\chi }=-2\sum \left[A_{n}{\tilde{R}}^{n}+\left(\frac{n}{n+1}A_{n}+D_{n}\right){\tilde{R}}^{-\left(n+1\right)}\right]P_{n}^{m}\left(\mu \right)e^{im\varphi } \end{array}$$

where $K_{n}\left(\tilde{R}/\lambda \right)=K_{n+1/2}\left(\tilde{R}/\lambda \right)/{\tilde{R}}^{1/2}K_{n+1/2}\left(1/\lambda \right)$ and $K_{n+1/2}$ is the modified Bessel function.

Let us next assume that the polarized nano-particle (NP) is initially uncharged, implying that $C_{0}=0$. Enforcing the zero ion-flux boundary conditions $\frac{\partial }{\partial \tilde{R}}\left(2\bar{\phi }+\bar{Q}\right)=0$ across the NP surface [10,19], renders.

(4) $$\left[1-{\left(\lambda /\lambda_{0}\right)}^{2}\right]\frac{\partial \bar{Q}}{\partial \tilde{R}}+\frac{\partial \bar{\chi }}{\partial \tilde{R}}=0\;\mathrm{or}\;\left(n+1\right)D_{n}^{m}=i\Omega C_{n}^{m};\;\Omega =\frac{\omega a\lambda_{0}}{D}$$

where Ω represents the RC frequency, since for a relatively thin EDL $\lambda \approx \lambda_{0}$ and ${\frac{\partial }{\partial \tilde{R}}{Κ}_{n}\left(\tilde{R}/\lambda \right)|}_{\tilde{R}=1}\approx -a/\lambda_{0}$ [9,10].

**Figure 1:**
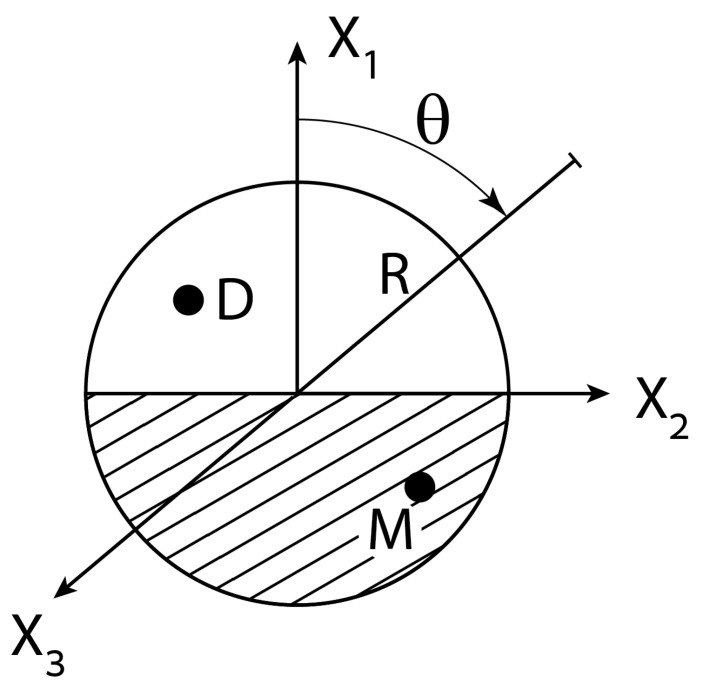
The interface between two homogenous hemispheres of a metallo-dielrctric (MD) Janus that is subjected to non-uniform ambient electric fields expressed in spherical coordinates (Equation (3b)).

The interface between the two homogenous hemispheres of the MD JP (Figure 1) is perpendicular to the $x_{1}$ axis, such that the surface of the metallic half sphere is given by 0≥μ≥−1, 0≤φ≤2π and that of the dielectric part by 1≥μ≥0, 0≤φ≤2π. It is also assumed that on the dielectric (vanishing small permittivity) surface, the induced-charge density (see Equation (4a)) is practically null and thus $\frac{\partial \bar{\chi }}{\partial \tilde{R}}=0$. On the other hand, the polarized metallic surface (infinitely large permittivity) is considered as equipotential, which suggests following Equations (2) and (3) that on the metallic (coated) part, $\bar{Q}=\bar{\chi }$ and $\frac{\partial \bar{Q}}{\partial \tilde{R}}=-\bar{Q}\left(a/\lambda_{0}\right)$ (thin EDL).

Finally, substituting these relations back into Equation (4a) renders the Robin-type boundary conditions $\frac{\partial \bar{\chi }}{\partial \tilde{R}}=-i\Omega \bar{\chi }$ on the metallic surface, which is the common boundary condition enforced on an ideally polarizable particle in terms of its surface capacitance [17].

In order to solve the following mixed boundary problem:

(5) $$\frac{\partial \bar{\chi }}{\partial R}=\left\{\begin{array}{c} -i\Omega \bar{\chi }\\ 0 \end{array}\right\}\left\{\begin{array}{c} 0\geq \mu \geq -1\\ 1\geq \mu \geq 0 \end{array}\right\}$$

with $\bar{\chi }$ given in Equation (3), we apply the Fourier—Legendre methodology [45], by multiplying Equation (5) by $P_{l}^{m}\left(\mu \right)$ and integrating the resulting expression over the surface of each hemisphere. Using the Legendre orthogonal properties leads to:

(6) $$\left[2\left(n+1\right)-i\Omega \right]D_{n}^{m}=i\Omega \frac{2n+1}{n+1}A_{n}^{m}-i\Omega \frac{\left(2n+1\right)\left(n-m\right)!}{(n+m)!}\sum_{l\neq n}^{}\left[\left(\frac{2l+1}{l+1}\right)A_{l}^{m}+D_{l}^{m}\right]\gamma_{l,n}^{m}$$

where

(7) $$\gamma_{l,n}^{m}=\int_{0}^{1}P_{l}^{m}\left(\mu \right)P_{n}^{m}\left(\mu \right)d\mu .$$

Equation (6) is the sought relation for evaluating the various multipole coefficients $D_{n}^{m}$ in (3b) depending on the prescribed amplitude coefficients $A_{n}^{m}$ of the non-homogenous ambient field Equation (1). Once Equation (6) is solved to determine $D_{n}^{m}$ in terms of the dimensionless RC forcing frequency Ω, the induced-charge coefficients $C_{n}^{m}$ in (3a) can be directly found from (4b). It is important to note here that Equation (6) was obtained for a spherical MD JP that is exposed to an arbitrary AC non-uniform applied field. In the DC limit (Ω=0), we readily find from (4b) that $D_{n}^{m}=0$, which is a consequence of imposing the Neumann boundary condition $\frac{\partial \bar{\chi }}{\partial \tilde{R}}=0$ on $\bar{R}=1$ for 1≥μ≥−1 and of the special form selected for the exterior potential in (3b). Furthermore, it is also worth mentioning that Equation (6) can be readily reduced to the corresponding relation, which holds for a (full) *homogenous* metallic spherical NP by letting in Equation (6):

(8) $$\gamma_{e,n}^{m}=\frac{1}{2}\int_{-1}^{1}P_{e}^{m}\left(\mu \right)P_{n}^{m}\left(\mu \right)d\mu =\frac{(n+m)!}{\left(2n+1\right)\left(n-m\right)!}\delta_{mn}$$

Thus, in the DC limit, substituting Equation (8) into Equation (7) simply renders by virtue of (4a):

(9) $$\left[\left(n+1\right)-i\Omega \right]D_{n}^{m}=i\Omega \frac{2n+1}{n+1}A_{n}^{m};\;\left[\left(n+1\right)-i\Omega \right]C_{n}^{m}=\left(2n+1\right)A_{n}^{m}.$$

We conclude this section by bearing in mind that the present analysis holds both for a homogeneous ideally polarized particle (NP) as well as for a Janus spherical colloid (JP), subject to either DC or AC excitations of arbitrary non-uniform applied electric fields. In the case of a JP, the corresponding higher-order multipoles ($D_{n}^{m}$) can be found by inverting Equation (6), whereas for an homogeneous particle (NP), they are given explicitly by (9) in terms of the prescribed forcing amplitudes $A_{n}^{m}.$ Next we will consider few dipolophoretic (DIP) DC and AC scenarios, involving both NP and JP under different non-uniform electric excitations. In particular, we will separately discuss cases of dielectrophresis (DEP) and induced-charge electrophoresis (ICEP), involving either NP or JP under general 3D travelling—wave harmonic forcing (linear and helical) in connection with evaluating the DIP dynamical loads (force and torque) exerted on the freely suspended particle.

## 3. DEP

### 3.1. Theoretical Background

Due to the spatial non-uniformity of the ambient electric field, the particle (regardless of its polarization) will experience a DEP force, resulting in a finite phoretic motion. The DEP force depends on the multipole system within the colloid and on the partial derivatives of the applied field evaluated at the locations of these higher-order singularities inside the particle. In particular and following the notations of [19], the general DEP force acting on the NP under non-uniform AC excitation can be expressed as:

(10) $${\vec{F}}_{DEP}=-\pi \left(\frac{\partial }{\partial x_{1}},\frac{\partial }{\partial x_{2}},\frac{\partial }{\partial x_{3}}\right)\sum_{s}G_{\alpha \beta \gamma }^{*}{\left(-1\right)}^{\alpha +\beta +\gamma }\frac{\partial^{\alpha +\beta +\gamma }}{\partial x_{1s}^{\alpha }\partial x_{2s}^{\beta }\partial x_{3s}^{\gamma }}\left({\bar{\chi }}_{am}\right)$$

where the upper (*) stands for the complex conjugate, (α,β,γ) are positive integers such that $G_{\alpha \beta \gamma }$ represent higher-order multipoles of order (the familiar n=α+β+γ dipole term corresponds to denotes summation over the positions of all $\sum_{S}$) and n=1 internal multipoles located at ($x_{1s}^{},x_{2s}^{},x_{3s}^{}$). Next, we note following Equations (11) and (3) that $\bar{\chi }=2\bar{\chi^{\prime }}+2{\bar{\chi }}_{am}$, where the disturbance potential is given by

(11) $$\bar{\chi^{\prime }}=-\sum_{n}\sum_{m}{\tilde{D}}_{n}^{m}R^{-\left(n+1\right)}P_{n}^{m}\left(\mu \right)e^{im\varphi }=-\sum_{s}G_{\alpha \beta \gamma }{\left(-1\right)}^{\alpha +\beta +\gamma }\frac{\partial^{\alpha +\beta +\gamma }}{\partial x_{1s}^{\alpha }\partial x_{2s}^{\beta }\partial x_{3s}^{\gamma }}\left(\frac{1}{R}\right).$$

Here, $R^{2}=\sum_{i=1}^{3}{\left(x_{i}-x_{is}\right)}^{2}$ and ${\tilde{D}}_{n}^{m}=\frac{n}{n+1}A_{n}^{m}+D_{n}^{m}$. Equation (11) can be used (as shown below) to determine the multipole outputs $G_{\alpha \beta \gamma }$ in terms of the ambient amplitudes $A_{n}^{m}$ and the coefficients $D_{n}^{m}$ arising from solving the electrostatic problem around the NP.

Since we use concurrently two coordinate systems (spherical & Cartesian) with common origin at the NP center, it is useful to employ the following relations between external and internal spherical harmonics expressed in the two systems [48]:

(12) $$P_{n}^{m}\left(\mu \right)R^{-\left(n+1\right)}e^{im\varphi }=\frac{{\left(-1\right)}^{n}}{\left(n-m\right)!}\frac{\partial^{n-m}}{\partial x_{1}^{n-m}}{\left(\frac{\partial }{\partial x_{2}}+i\frac{\partial }{\partial x_{3}}\right)}^{m}\left(\frac{1}{R}\right)$$

and

(13) $$\underset{R\to 0}{\lim}\left\{\frac{\partial^{n-m}}{\partial x_{1}^{n-m}}{\left(\frac{\partial }{\partial x_{2}}+i\frac{\partial }{\partial x_{3}}\right)}^{m}\left(R^{n}P_{n}^{m}\left(\mu \right)e^{im\varphi }\right)\right\}=\left\{\begin{array}{c} n!\\ \frac{1}{2}\left(n+m\right)! \end{array}\right\}\;\mathrm{for}\;\left\{\begin{array}{c} m=0\\ m\neq 0 \end{array}\right\}.$$

The above procedure will be first demonstrated for an axisymmetric electric forcing whereby m=0.

### 3.2. Axisymmetric Forcing

Consider for example the JP depicted in Figure 1 which is subjected to axisymmetric AC ambient fields expressed in cylindrical coordinates $\left(x_{1},r\right)$, $e^{i\left(kx_{1}-\omega t\right)}I_{0}\left(kr\right)$ or $e^{\pm \lambda x_{1}-i\omega t}J_{0}\left(\lambda r\right)$, where $I_{0}$ and $J_{0}$ denote the common Bessel functions. Both potentials exhibit azimuthal symmetry with respect to the axial $x_{1}$ axis, which is chosen later in the direction of gravity. The first potential describes a simple travelling- wave $e^{i\left(kx_{1}-\omega t\right)}$ with a wave number k, moving along the $x_{1}$ axis (r=0) with a velocity $\omega /k_{1}$. The second potential corresponds to the induced potential in a (vertical) cylindrical pore of radius b bounded by two circular electrodes; say one powered and other grounded. The Eigen-functions λ depend on the specific boundary conditions applied on the cylindrical wall. For the case of insulating cylindrical walls, λ are determined by $J_{1}\left(\lambda b\right)=0$ and for conducting walls by $J_{0}\left(\lambda b\right)=0$. As shown in the sequel, the particular form of the applied potential uniquely determines the ambient amplitudes $A_{n}$ in Equation (1).

Taking advantage of axial symmetry, we note that the induced DEP force acts along the $x_{1}$ axis. Furthermore, the multipoles $G_{n00}$ in Equation (10) can be expressed in this case according to Equations (11) and (12) (see also [19]) simply as $G_{n00}=\frac{{\tilde{D}}_{n}}{n!}$. Finally, substituting Equation (13) for m=0 in Equation (10) and using Equation (1), renders the following rather compact expression for the axial DEP force (Re denotes the real part):

(14) FDEP(1)=−2πRe∑n=1[nAn+(n+1)Dn]An+1*

Thus, we may conclude that Equation (14) provides the sought expression for the DEP force acting on a spherical NP exposed to a non-uniform AC forcing. In the particular DC limit (Ω=0), we can deduce following (4b) that $D_{n}=0$ and thus Equation (14) is reduced to the following rather simple quadratic expression (valid to both NP and JP):

(15) $$F_{DEP}^{\left(1\right)}=-4\pi \sum_{n=1}nA_{n}A_{n+1}^{}.$$

It can be readily verified that the above general DC DEP solution agrees with that given in Equation (24) of [9]. As far as the AC case is considered, one can examine for example the ‘constant gradient’ approximation for a NP [7,8,9,17], where $D_{1}=\frac{3}{2}\frac{i\Omega }{\left(2-i\Omega \right)}A_{1}$ and $\left(A_{1},A_{2}\right)$ are non-zero and real coefficients, yielding $F_{DEP}^{\left(1\right)}=4\pi A_{1}A_{2}\left(\Omega^{2}-2\right)/\left(\Omega^{2}+4\right)$ in agreement with Equation (17) of [17]. 

Scrutiny of the above relations reveal that the DEP force (for spherical NP’s), arises only due to interactions between neighboring modes, i.e., one even and the other odd. Clearly, if all modes of the ambient field Equation (1) are either even or odd, the resulting DEP force exerted on an homogenous NP is null. However, the situation for a JP is quite different as demonstrated below. Let us next discuss several DEP cases involving DC forcing and then the more intriguing cases of AC excitations.

#### 3.2.1. Standing Wave

**Figure 2:**
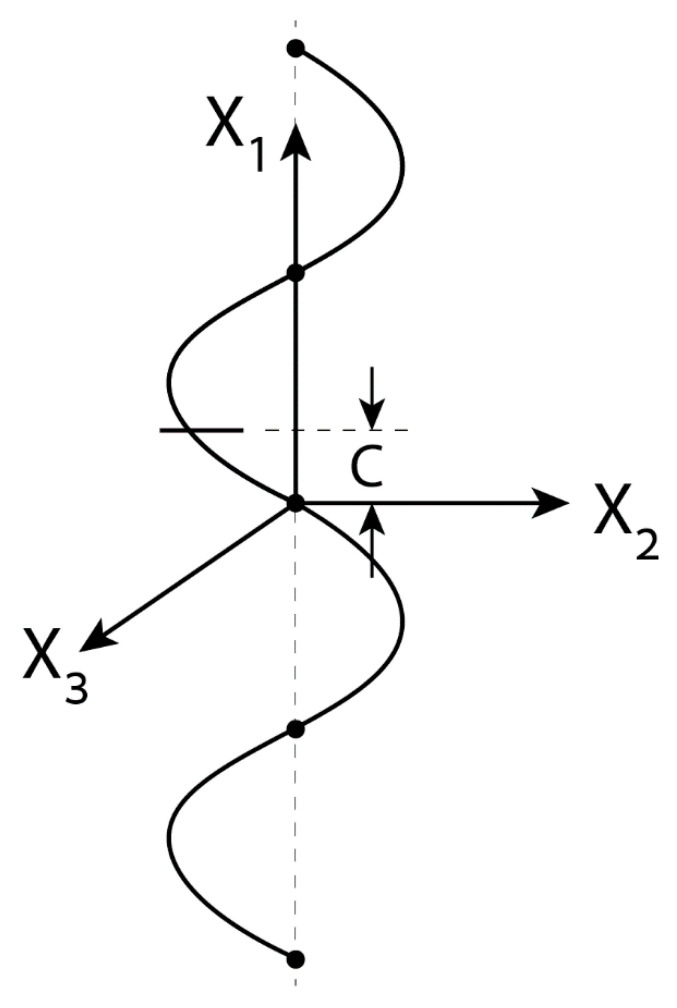
A free polarizable micro/nano NP or JP lying on the $x_{1}$ axis (r=0) at a distance c from the nearest nodal point of a standing wave.

Expressed in a cylindrical coordinate system ($x_{1},r$) placed at the origin of the spherical NP, the ambient electric potential of a harmonic standing wave of unit amplitude and wave length 2π/k, can be written as,

(16) $${\bar{\chi }}_{am}=e^{ik\left(x_{1}-c\right)}I_{0}\left(kr\right)\;;\;x_{2}^{2}+x_{3}^{2}=r^{2}$$

where *c* denotes the distance (Figure 2) between the center of the spherical colloid lying on the $x_{1}$ axis (r=0) to the nearest node of the standing wave, where $k\left(x_{1}-c\right)=n\pi ,n=1,2,3,$…. Clearly, for c=0, the colloid is placed precisely at one of the wave nodal points.

Expanding both Equations (1) and (16) in a Taylor series in terms of $x_{1}$, evaluated near the origin and along the axis of symmetry ($r=0,\;\tilde{R}=x_{1}/a,\;m=0$), readily implies by analytic continuation that:

(17) $$A_{n}=-e^{-ikc}\frac{{\left(ika\right)}^{n}}{n!}$$

since $P_{n}\left(1\right)=I_{0}\left(0\right)=1$. Finally, substituting Equation (17) into Equation (15) yields:

(18) FDEP(1)=4π(ka)3Re{ie−2ikc∑n=1∞(−1)n(ka)2(n−1)(n−1)!(n+1)!}=4π(ka)J2(2ka)sin(2kc)

Note that Equation (18) is exact and shows that when subjected to a DC standing wave excitation, the DEP acting on a spherical NP is null when c=0 (i.e., colloid placed at a wave nodal point), or obviously under the long wavelength approximation (uniform field), whereby ka→0.

#### 3.2.2. Cylindrical Pore

**Figure 3:**
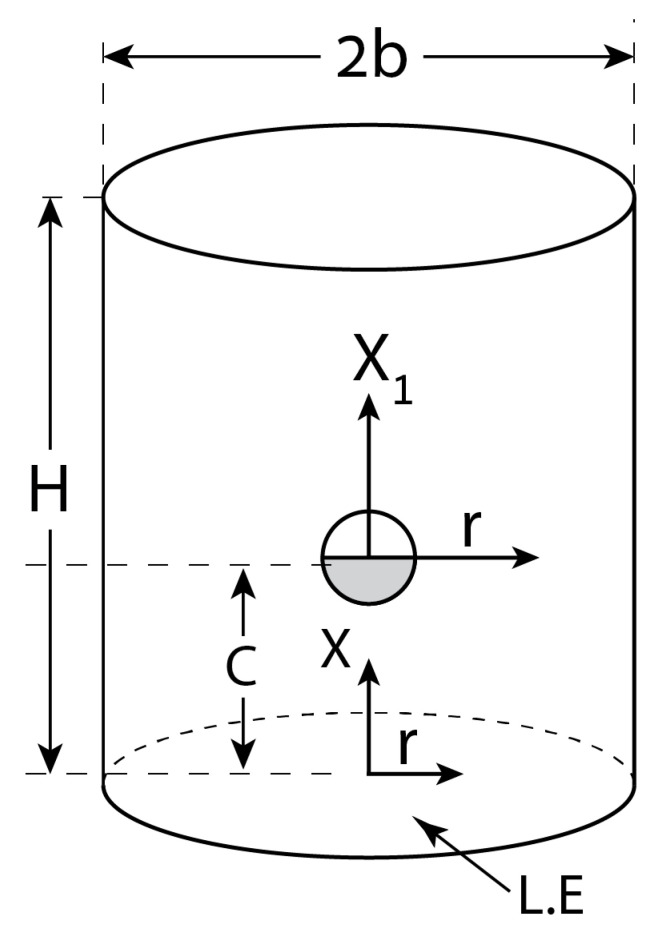
Freely suspended spherical colloid (NP/JP) placed at a distance *c* above a powered circular electrode along the axis of a cylindrical pore (r=0) of radius *b* and height *H*.

Here, we consider the case of a free spherical colloid (including JP) placed at a distance *c* above a powered circular electrode along the axis of a cylindrical pore (r=0) of radius *b* and height *H* (see Figure 3). The corresponding electrical potential expressed in a cylindrical coordinate system attached to the colloid is:

(19) $${\bar{\chi }}_{am}=\frac{\sinh\left[\lambda \left(H-x_{1}-c\right)\right]}{\sinh\left(\lambda H\right)}J_{0}\left(\lambda r\right)$$

such that the lower electrolyte ($x_{1}=-c$) is powered with a potential $J_{0}\left(\lambda r\right)$, whereas the upper $\left(x_{1}=H-c\right)$ electrode is grounded. If the cylindrical wall of the pore is taken as insulating, then λ (the lowest eigenvalue) is determined by imposing $J_{1}\left(\lambda b\right)=0$, namely λb=3.8317, whereas if the wall is conducting $J_{0}\left(\lambda b\right)=0$, then λb=2.408. Expanding both Equations (1) and (19) in a Taylor series around the origin $\left(r=0,\;\tilde{R}=x_{1}/a,\;m=0\right)$, renders:

(20) $$A_{n}=\frac{{\left(-1\right)}^{n+1}}{n!}\frac{{\left(\lambda a\right)}^{n}}{\sinh\left(\lambda H\right)}\left\{\begin{array}{c} \cosh\lambda \left(H-c\right)\\ \sinh\lambda \left(H-c\right) \end{array}\right\};\;\left\{\begin{array}{c} n-odd\\ n-even \end{array}\right\}.$$

Finally, substituting Equation (20) into Equation (15) yields:

(21) $$F_{DEP}^{\left(1\right)}=\frac{2\pi {\left(\lambda a\right)}^{3}\sinh\left[2\lambda \left(H-c\right)\right]}{\sinh^{2}\left(\lambda H\right)}\sum_{n=1}^{}\frac{{\left(\lambda a\right)}^{2(n-1)}}{\left(n-1\right)!\left(n+1\right)!}=2\pi \left(\lambda a\right)I_{2}\left(2\lambda a\right)\frac{\sinh\left[2\lambda \left(H-c\right)\right]}{\sinh^{2}\left(\lambda H\right)}$$

Again, the right-hand side of Equation (21) is exact, showing that under an ambient non-uniform electric potential Equation (19), a metallic homogenous NP situated in a circular pore r=0, λ≠0), will always experience a DEP force depending on the geometric parameters (*b*, *H*) and the colloid position (*c*). Nevertheless, we see that for λ→0, Equation (19) reduces to a linear field (in $x_{1}$) for which the DEP force Equation (21) vanishes. 

As far as we understand, these analytic (closed form) expressions for the DEP force experienced by a spherical colloid under DC axisymmetric non-uniform forcing, are given here for the first time. In the next section, we will demonstrate how the foregoing analysis can be extended for non-symmetric (azimuthal) electric forcing.

### 3.3. Non-Symmetric Forcing

As an example of an *asymmetric* ambient potential expressed in a cylindrical coordinate system ($x_{1},r,\varphi )$, let us consider the case of a general *helical* (spiral) wave- field given by $e^{i\left(kx-\omega t+m\varphi \right)}I_{m}\left(kr\right)$, where k (as before) represents the wave number and *m* is taken here as a positive integer. The particular choice of m=0 clearly corresponds to the previously discussed case of an axisymmetric (rectilinear) sinusoidal-wave along the $x_{1}$ axis. The special choice of m=1 is also of special interest, since it describes a non-uniform rotating wave field (ROT), which is further analyzed in the sequel. It is worth mentioning that the above (Laplace) electric field, can be considered as a limiting case of the following optical (Helmholtz) Bessel beam of TM polarization $e^{i(k_{x}x-\omega t+m\varphi )}J_{m}(k_{r}r)$ whereby $k_{x}^{2}+k_{r}^{2}=k_{0}^{2}$ in the limit where the wavelength $k_{0}$ is vanishingly small, implying that $k_{r}^{}=\pm ik_{x}$. Again, the case of m=0 (corresponds optical analogy) to linear polarization whereas that of m=1 to a circular polarization [49].

A general wave field contains an infinite number of modes $A_{n}^{m}\left(1\right)$, where *n* is a positive integer and m=0,1,2,…,n. In the case of axisymmetric forcing (m=0), it has been shown that the DEP force results from interactions between neighboring (i.e., even and odd) modes, namely $A_{n}A_{n+1}$. By using symmetry arguments, one finds that this force acts only along the axis of symmetry. However, for m≠0, the resulting DEP force has also finite components along the transverse axes ($x_{2},x_{3}$). These terms arise from a $A_{n}^{m}A_{n+1}^{m\pm 1}$- type interaction [50].

In order to keep the analysis tractable and for illustrating the general approach, let us select m=1 and arbitrary *n*. Such a case is of particular interest, since it generalizes the common [4,11] ROT (electro-rotation) AC forcing (under the long-wave approximation), which will be discussed in the sequel. It should be noted however that for a single *m*-mode, the DEP force along the transverse ($x_{2},x_{3}$) axes is null, but still there is a finite DEP force acting in the axial ($x_{1}$) direction. Thus, we refer to the ambient asymmetric wave field in the form of Equation (1):

(22) $${\bar{\chi }}_{am}\left(R,\theta ,\varphi \right)=-\sum_{n=1}^{\infty }A_{n}^{1}R^{n}P_{n}^{1}\left(\mu \right)e^{i\varphi }$$

where $A_{n}^{1}$ are prescribed complex amplitudes. Following Equations (11) and (13), we can express the disturbance potential as:

(23) $${{\bar{\chi }}^{\prime }}_{}=-\sum_{n=1}^{\infty }{\tilde{D}}_{n}^{1}P_{n}^{1}\left(\mu \right)R^{-\left(n+1\right)}e^{i\varphi }=-\sum_{n=1}^{\infty }\frac{{\tilde{D}}_{n}^{1}}{\left(n-1\right)!}\frac{\partial^{n-1}}{\partial x_{1}^{n-1}}\left(\frac{\partial }{\partial x_{2}}+i\frac{\partial }{\partial x_{3}}\right)\left(\frac{1}{R}\right)$$

which implies that the corresponding multipoles in Equations (10) and (11) are simply given by $G_{n-1,1,0}=G_{n-1,0,1}={\tilde{D}}_{n}^{1}/\left(n-1\right)!$. Using the same relations and Equation (13), one also gets:

(24) $$\underset{R\to 0}{\lim}\left\{\frac{\partial^{n-1}}{\partial x_{1}^{n-1}}\left(\frac{\partial }{\partial x_{2}}+i\frac{\partial }{\partial x_{3}}\right)\left(R^{n}P_{n}^{1}\left(\mu \right)e^{i\varphi }\right)\right\}=\frac{1}{2}\left(n+1\right)!$$

Finally, substituting Equation (24) into Equation (10), renders the following expression for the axial DEP force exerted on a spherical NP under *asymmetric* (m=1) AC excitation:

(25) FDEP(1)=−πRe∑n=1n(n+2)[nAn1+(n+1)Dn1]An+1*1

Clearly, under DC forcing (ω=0), the coefficients $D_{n}^{1}$ are null by virtue of (4b) and thus Equation (25) is simply reduced to:

(26) $$F_{DEP}^{\left(1\right)}=-2\pi \sum_{n=1}n^{2}\left(n+2\right)A_{n}^{1}A_{n+1}^{1}$$

Below we analyze few cases of non-uniform asymmetric wave forcing.

### 3.4. Helical Wave

In a similar manner to Equation (16), let us consider a helical (circumferential) wave (m=1) propagating along the $x_{1}$ axis with a linear velocity ω/k. For the particular case, where the position of the colloid on the axis of symmetry is displaced at a distance *c* from the nearest wave mode, one can express the ambient potential in a body-attached coordinate system as:

(27) $${\bar{\chi }}_{am}=e^{i\left[k\left(x_{1}-c\right)+\varphi -\omega t\right]}I_{1}\left(kr\right)=-\sum_{n=1}^{\infty }A_{n}^{1}{\tilde{R}}^{n}P_{n}^{1}\left(\mu \right)e^{i\varphi }$$

Expanding Equation (27) in a Taylor series near the origin (colloid center) in powers of $x_{1}$ $\left(\tilde{R}=x_{1}/a\right)$) for r=0 (μ=1), by recalling that $I_{1}\left(kr\right)\to \frac{1}{2}\sqrt{1-\mu^{2}}kx_{1}$, $P_{n}^{1}\left(1\right)\to \frac{1}{2}\sqrt{1-\mu^{2}}n\left(n+1\right)$ and $e^{ikx}=\sum_{n=0}\frac{{\left(ikx\right)}^{n}}{n!}$, yields the following explicit expression:

(28) $$A_{n}^{1}=-\frac{i^{n-1}e^{-ikc}}{\left(n+1\right)!}{\left(ka_{}\right)}^{n},\;\;n\geq 1.$$

where the frequency dependent term has been suppressed. Finally, substituting Equation (28) into Equation (26) renders for the steady DEP force (ω=0):

(29) FDEP(1)=−2π(ka)3Re{ie−2ikc∑n=1∞(−1)n(n(n+1)!)2(ka)2(n−1)}=π2sin(2kc)ζ2ddζ{ζddζ[J0(2kζ)−1ζ2]};

where ζ=ka. It is again worth mentioning that if the particle lies at one of the nodal points of the spiral wave, namely when 2kc=nπ, the axial DEP force Equation (29) vanishes and reaches a maximum value for 2kc=(n+1/2)kc.

Extending the analysis for AC forcing (ω≠0), is straightforward when using the additional multipole term related to $D_{n}^{1}$ in Equation (26). Note however that under AC excitations and for a homogenous NP, $D_{n}^{m}$ are given explicitly in terms of the amplitudes $A_{n}^{m}$ of the ambient field (9a) and the RC frequency Ω. The same expression also applies for a JP where $D_{n}^{m}$ is determined by solving Equation (7).

Before concluding this section, which provides a general methodology for evaluating the DEP forces and presenting next the corresponding ICEP analysis, it is appropriate to discuss the recent DEP results on traveling- wave of metallic NP’s reported in [27]. Flores-Mena et al. [27], considered a special axisymmetric two-mode travelling wave excitation (c=0) given by $-e^{i\left(kx-\omega t\right)}I_{0}\left(kr\right)$, for which case Equation (17) renders $A_{1}=ika,\;A_{2}=-{\left(ka\right)}^{2}/2$. We also observe from Equation (9a) that $D_{1}=\frac{3}{2}i\Omega /\left(2-i\Omega \right)$ and thus, since according to Equation (14) FDEP(1)=−2πRe{(A1+2D1)A2*}, we finally obtain $F_{DEP}^{\left(1\right)}=-6\pi \left(ka\right)\Omega /\left(4+\Omega^{2}\right)$, which coincides with Equation (25) of [27]. Nevertheless, aside from being valid only for the particular case where c=0, this expression should be considered only as the leading-order ‘weakly’ non-uniform field (long- wavelength) approximation (i.e., ka<<1) of the exact expression found by substituting Equations (17) and (9a) into Equation (14), namely:

(30) FDEP(1)=−2πRe{∑n=1∞(n+1)DnAn+1*}=−2π(ka)3Ω∑n=1∞(2n+1)(ka)2(n−1)(n!)2[(n+1)2+Ω2]

The above *exact* expression can be also easily extended for the more general ‘displaced’ case, namely c≠0. The DEP result of [27] is indeed seen to be the leading term (n=1) of the infinite summation Equation (30).

## 4. ICEP

### 4.1. General Formulation

In addition to the DEP force, which arises because of the non-uniformity of the ambient electric field, the free colloid also experiences an ICEP force due to the flow field driven by the induced-charge electro-osmosis in the surrounding solute. The total force (DEP + ICEP) exerted on the NP, is referred to as dipolophoresis (DIP), where in general DEP and ICEP act (especially at low frequencies) in different directions [8,9,17]. Following Teubner’s [47] formulation and using Lorentz reciprocal relation, the ICEP force acting on a freely suspended polarizable particle subject to an AC field, can be written [3] as:

(31) $$F_{ICEP}^{\left(i\right)}=-\frac{1}{8\lambda_{0}^{2}}\int_{\forall }{\bar{Q}}^{*}\left(u_{j}^{\left(i\right)}-\delta_{ij}\right)\frac{\partial \bar{\chi }}{\partial x_{j}}d\forall$$

where $\lambda_{0}^{}$ is the Debye length scale (EDL), ${\bar{Q}}^{*}$ (conjugate) is the induced charge density Equation (3a) and $\bar{\chi }$ (harmonic) is the electric potential Equation (3). In addition, ∀ represents the semi-unbounded fluid volume and $u_{j}^{\left(i\right)}$ is a generic solution of the homogenous Stokes equation [51]. In particular, $u_{j}^{\left(i\right)}$ denotes the i-*th* components of the Stokesian velocity induced in the fluid due to unit velocity of a rigid particle moving along the *j* axis. For example, for a spherical colloid, one has [51]:

(32) $$u_{j}^{\left(i\right)}-\delta_{ij}=\left(\frac{3}{4\tilde{R}}+\frac{1}{4{\tilde{R}}^{3}}-1\right)+\frac{3}{4}\frac{x_{i}x_{j}}{{\tilde{R}}^{2}}\left(\frac{1}{\tilde{R}}-\frac{1}{{\tilde{R}}^{3}}\right)$$

Making use of the fact that $\lambda_{0}/a\to 0$ (thin EDL) and $u_{j}^{\left(i\right)}=\delta_{ij}$ ($\delta_{ij}$ being the Kronecker delta tensor) on the colloid surface *S*, Equation (32) together with Equation (33) can be reduced to the following surface integral, since $\bar{Q}$ decays exponentially away from the colloid as $e^{-\left(R-a\right)/\lambda_{0}}$:

(33) $$F_{ICEP}^{\left(i\right)}=-\frac{1}{8}{\int_{S}{\bar{Q}}^{*}\frac{\partial \bar{\chi }}{\partial x_{j}}\frac{\partial }{\partial R}\left(u_{j}^{\left(i\right)}-\delta_{ij}\right)|}_{\tilde{R}=1}dS=\frac{3}{16a}\int_{S}{\bar{Q}}^{*}\left(\frac{\partial \bar{\chi }}{\partial x_{i}}-n_{i}\frac{\partial \bar{\chi }}{\partial R}\right)dS+O\left(\lambda_{0}/a\right)$$

where $n_{i}=x_{i}/R$ denotes the external normal (unit) vector to *S*.

### 4.2. Axisymmetric Traveling Wave

Let us first consider the axial ICEP force exerted on a spherical colloid under a non-uniform axisymmetric AC loading, by noting that ${\frac{\partial \bar{\chi }}{\partial x_{1}}-n_{1}\frac{\partial \bar{\chi }}{\partial R}|}_{\tilde{R}=1}=\frac{1}{a}\left(1-\mu^{2}\right)\frac{\partial \bar{\chi }}{\partial \mu }$. Next, employing this relation together with Equation (3) and the following identity [48]:

(34) $$\left(2n+1\right)\left(1-\mu^{2}\right)\frac{dP_{n}\left(\mu \right)}{d\mu }=n\left(n+1\right)\left[P_{n-1}\left(\mu \right)-P_{n+1}\left(\mu \right)\right]$$

in the second surface integral in Equation (33) and using orthogonality, finally yields:

(35) FICEP(1)=3πRe{∑n=1n(n+1)2n+1[Cn−1*2n−1−Cn+1*2n+3](An+D˜n)}; D˜n=nn+1An+Dn.

The above is the sought expression for the axial ICEP force exerted on a freely suspended spherical particles (including JP) that is exposed to an arbitrary non-uniform axisymmetric AC electric forcing. Considering for example, the case of a perfectly polarized (metallic) NP. Imposing the equipotential boundary condition $\bar{\phi }=0$ on $\bar{R}=1$ and noting that according to Equations (2) and (3) $C_{n}=A_{n}+{\tilde{D}}_{n}$ (thin EDL), Equation (35) is simply reduced to:

(36) FICEP(1)=6πRe{∑n=1(n+1)(2n+1)(2n+3)CnCn+1*}.

Clearly, in the DC limit, Equation (36) renders [9]:

(37) $$F_{ICEP}^{\left(1\right)}=12\pi \sum_{n=1}\frac{\left(n+1\right)}{\left(2n+1\right)\left(2n+3\right)}C_{n}C_{n+1}=12\pi \sum_{n=1}\frac{A_{n}A_{n+1}}{n+2}$$

since under DC Equation (3), $D_{n}=0$ and $C_{n}=\frac{2n+1}{n+1}A_{n}$.

Next, combining Equation (37) with Equation (15), readily yields the final expression for the total DIP (DEP + ICEP) force acting on a metallic spherical particle under a steady (DC) non-uniform axisymmetric forcing:

(38) $$F_{DIP}^{\left(1\right)}=-4\pi \sum_{n=1}^{\infty }\frac{\left(n-1\right)\left(n+3\right)}{n+2}A_{n}A_{n+1}$$

in agreement with [9]. It is rather interesting to observe [6,7,8] that by considering only the first two modes of the ambient field (‘constant gradient’ approximation), namely keeping only $A_{1}$ and $A_{2}$(n=1) in Equation (38), the corresponding DIP force for a spherical NP vanishes, since the DEP and ICEO components are equal but act in opposite directions. However, as demonstrated in [9,10] and in the sequel, this intriguing result is true only under the limit of an infinitely thin EDL and DC forcing.

In the case of an AC ambient axisymmetric non-uniform fields, the coefficients $C_{n}$ can be readily found for a metallic NP by realizing that in addition to the surface *S* being an equipotential (ϕ=0), one has to satisfy following (4a) $\left(n+1\right)D_{n}=i\Omega C_{n}$ and $C_{n}=A_{n}+{\tilde{D}}_{n}$, so that:

(39) $$C_{n}=\frac{\left(2n+1\right)A_{n}}{\left(n+1\right)-i\Omega },\;\;\;D_{n}=\frac{(2n+1)i\Omega A_{n}}{\left(n+1)[(n+1)-i\Omega \right]}.$$

Substituting Equation (39) in Equation (37) lastly renders:

(40) FICEP(1)=6πRe{∑n=1(n+1)AnAn+1*[(n+1)−iΩ][(n+2)+iΩ]}.

which reduces to (37b) for Ω=0.

For the purpose of illustration, let us consider the phasor of the previously discussed axisymmetric travelling wave excitation (c=0), namely $-e^{ikx_{1}}I_{0}\left(kr\right)$, for which case we have $A_{n}=\frac{{\left(ika\right)}^{n}}{n!}$. Substituting these amplitudes back into Equation (40) leads to:

(41) $$F_{ICEP}^{\left(1\right)}=6\pi \Omega {\left(ka\right)}^{3}\sum_{n=1}\frac{{\left(ka\right)}^{2(n-1)}}{[{\left(n+1\right)}^{2}+\Omega^{2}][{\left(n+2\right)}^{2}+\Omega^{2}]{\left(n!\right)}^{2}}.$$

Note that if we consider only the first (leading) term in Equation (40), namely n=1, the infinite sum in Equation (41) simply reduces to $\Omega /\left[4+\Omega^{2}\right]\left[9+\Omega^{2}\right]$, which coincides with Equation (27) of [27]. Similarly, for the ‘constant gradient’ type forcing [7,8,9,17], where the only two (real) surviving coefficients in Equation (40) are ($A_{1},A_{2}$) one gets $F_{ICEP}^{\left(1\right)}=12\pi (6+\Omega^{2})A_{1}A_{2}/[(4+\Omega^{2})(9+\Omega^{2})]$, which again agrees with Equation (16) of [17].

### 4.3. Asymmetric Helical Wave

Here, we wish to determine the ICEP force acting on a spherical NP placed along the axis of a helical (spiral) wave ${\bar{\chi }}_{am}=-\frac{2}{ka}e^{i\left(kx-\varphi -\omega t\right)}I_{1}\left(kr\right)$. Following Equation (27), the corresponding amplitudes $A_{n}^{1}$ of such a circumferential wave-field are given for c=0 by $A_{n}^{1}=\frac{2{\left(ika\right)}^{n-1}}{\left(n+1\right)!}\;,\;(n>1)$. A homogenous NP or JP with its two-phase interface lying in the $x_{1}=0$ plane (Figure 1) will experience no ICEP force along the transverse axes ($x_{1},x_{2}$) since this helical field (m=1) is uniform in the transverse directions. Nevertheless, there is still a finite ICEO force acting in the axial $\left(x_{1}\right)$ direction, given (for thin EDL) by Equation (35), where $\bar{Q}$ and $\bar{\chi }$ are furnished by Equation (3) with m=1.

In order to evaluate the resulting ICEP surface integral in Equation (33), we make use of the following relation involving the corresponding Legendre polynomials [48]:

(42) $$\left(2n+1\right)\left(1-\mu^{2}\right)\frac{dP_{n}^{1}\left(\mu \right)}{d\mu }={\left(n+1\right)}^{2}P_{n-1}^{1}\left(\mu \right)-n^{2}P_{n+1}^{1}\left(\mu \right)$$

Substituting Equation (42) into Equation (35) and using orthogonally, finally yields:

(43) FICEP(1)=3π2Re{∑n=1n(n+1)2n+1[(n−1)(n+1)2n−1C*n−11−n(n+2)2n+3C*n+11](An1+D˜n1)}.

It is worth noting that Equation (43) holds for both metallic NP and JP. In the case of a perfectly conducting spherical (metallic) NP, one gets $A_{n}^{1}+{\tilde{D}}_{n}^{1}=C_{n}^{1}$ (since *S* is equipotential). Thus, for a freely suspended ideally polarized NP, placed under any AC axisymmetric (m=1) forcing, Equation (43) reduces to:

(44) FICEP(1)=3πRe{∑n=1n(n+1)(n+2)(2n+1)(2n+3)Cn1C*n+11}

The transverse DIP force is given by the sum of the DEP Equation (25) and ICEP Equation (44). In the DC limit, we readily get $F_{DIP}^{\left(1\right)}=-2\pi \sum_{n=1}n(n-1)(n+3)A_{n}^{1}A_{n+1}^{1}$. Thus, in the ‘constant gradient’ (linear) approximation, where only the first two terms ($A_{1}^{1\;}\;,A_{2}^{1}$) of the ambient field are kept, the transverse DIP (similar to the axial component Equation (38)) also vanishes regardless of the magnitude of the corresponding amplitudes. 

Under the present helical wave forcing Equation (39), $C_{n}^{1}=\frac{2\left(2n+1\right){\left(ika\right)}^{n-1}}{\left(n+1\right)!\left[n+1-i\Omega \right]}$ and the axial ($x_{1}$) ICEP component Equation (44) is given by,

(45) $$F_{ICEP}^{\left(1\right)}=6\pi (ka)\Omega \sum_{n=1}^{\infty }\frac{{\left(ka\right)}^{2(n-1)}}{\left(n-1\right)!\left(n+1\right)!\left[{\left(n+1\right)}^{2}+\Omega^{2}\right]\left[{\left(n+2\right)}^{2}+\Omega^{2}\right]}$$

which, as expected (compare for example to the corresponding DEP Equation (30)), is null both for a DC forcing (Ω=0) as well as under the long-wavelength approximation (ka→0), where the ambient field is uniform (i.e., ${\bar{x}}_{am}≃-x_{2}+ix_{3}$). This limiting case corresponds to the common rotating electric field (ROT) excitation [4], where $A_{n}^{1}=\delta \left(n-1\right)$ and n≥1. It is also worth noting, that for a homogenous spherical NP forced by ROT, both DEP and ICEP force components vanish and the polarized particle is only subjected to a finite DIP torque causing the NP to rotate around the $x_{1}$ axis.

## 5. DIP Torque

In order to complete the prevalent DIP formulation, we consider below the corresponding expressions for the ICEP and DEP torque components exerted on a spherical NP, which is exposed to a general helical wave field excitation, where $A_{n}^{1}=2\frac{{\left(ika\right)}^{n-1}}{\left(n+1\right)!}$. The electric potential and the induced charge density in the surrounding liquid phase, are given in Equation (3) as a summation over *n* for m=1. The ICEP torque, following Teubner’s [47] formulation (assuming thin EDL), can then be expressed, in a similar manner to Equation (34) by ($\bar{r}$ denotes the radius vector):

(46) $${\vec{T}}_{ICEP}=\frac{3}{8}\int_{S}{\bar{Q}}^{*}\left(\vec{r}\times \bar{\chi }\right)d\forall +O\left(\lambda_{0}/a\right)$$

Considering the real part of Equation (46), we deduce that the only torque component for the above helical wave field acts in the axial $x_{1}$ direction and is given by:

(47) TICEP(1)=6πIm{∑n=1n(n+1)2n+1Cn*1(An1+D˜n1)}

where Im denotes here the imaginary part.

Note however, that for a perfectly polarized NP, imposing the equipotential boundary conditions on *S* to Equation (2), implies that for an infinitely thin EDL $A_{n}^{1}+{\tilde{D}}_{n}^{1}=C_{n}^{1}$ and thus the ICEP torque Equation (47) is null, in agreement with the ROT result reported for (n=1) in [15]. It is also important to note, that Equation (47) is not restricted only for helical-waves and instead holds for any transverse excitation (m=1), providing $\lambda_{0}/a\to 0$. Thus, our conclusion that under the thin EDL limit, the ICEP torque vanishes for metallic spherical NP’s is quite general. Nevertheless, one should bear in mind that this is not necessarily true for a non-homogeneous spherical JP, as demonstrated in the sequel even under the thin EDL limit. Before considering this interesting JP case, we provide below for reasons of completeness, the corresponding expression for the DEP torque acting on a spherical NP under *asymmetric* (transverse) forcing.

Using the notation in Section 3.1, the DEP torque exerted on a free NP is given by,

(48) T→DEP=−πRe{∑SGαβγ*(−1)α+β+γ∂α+β+γ∂x1sα∂x2sβ∂x3sγ(r¯×χ¯am)}

For a spherical particle all multipoles are located at the origin and thus Equation (48) has to be evaluated at $\bar{r}=0$. Furthermore, making use of the following relation [48,50]

(49) $$\frac{P_{n}^{1}\left(\mu \right)e^{-i\varphi }}{R^{n+1}}=\frac{{\left(-1\right)}^{n}}{\left(n-1\right)!}\frac{\partial^{n-1}}{\partial x_{1}^{n-1}}\left(\frac{\partial }{\partial x_{2}}-i\frac{\partial }{\partial x_{3}}\right)\left(\frac{1}{R}\right)$$

implies that the multipoles in Equation (48) are given by $G_{n-1,1,0}=G_{n-1,0,1}=\frac{{\left(-1\right)}^{n}}{\left(n-1\right)!}{\tilde{D}}_{n}^{1}$ for n≥1, where ${\tilde{D}}_{n}^{1}=\frac{n}{n+1}A_{n}^{1}+D_{n}^{1}$ (see Equation (3)).

In the case of a helical wave field (m=1) acting on a spherical NP, symmetry arguments suggest that the resulting DEP torque has only one component around the $x_{1}$ axis. Thus, substituting Equation (49) into Equation (48) yields for $\bar{r}=0$,

(50) TDEP(1)=πIm{∑S(−1)n(n+1)!D˜n*1∂n−1∂x1n−1(∂∂x2+i∂∂x3)χ¯am}

Finally, recalling Equation (1) that ${\bar{\chi }}_{am}=-2\sum A_{m}^{1}R^{m}P_{m}^{1}\left(\mu \right)e^{-i\varphi }$ and using Equation (42), implies that

(51) $$\underset{R\to 0}{\lim}\frac{\partial^{n-1}}{\partial x_{1}^{n-1}}\left(\frac{\partial }{\partial x_{2}}+i\frac{\partial }{\partial x_{3}}\right)\left(R^{m}P_{m}^{1}\left(\mu \right)e^{-i\varphi }\right)\to 2\delta \left(m-n\right)$$

Finally, substituting Equation (51) in Equation (50), provides the sought expression of the axial DEP torque:

(52) T→DEP(1)=−4πIm{∑n=1(−1)n(n+1)!An1D˜n*1}

According to Equations (47) and (52) a metallic (perfectly symmetric) spherical NP, can experience a finite DIP torque only under AC excitations, since for Ω=0 the quadratic terms in these summations are real!

Consider for example a free homogenous NP, which is subjected to the same helical field (c=0) discussed in Section 4.3, where (28)$A_{n}^{1}=\frac{2{\left(ika\right)}^{n-1}}{\left(n+1\right)!}$. Equation (52) together with Equation (9) then render the following analytic expression:

(53) $$T_{DEP}^{\left(1\right)}=-16\pi \Omega \sum_{n=1}^{\infty }\frac{{\left(-1\right)}^{n}\left(2n+1\right)}{{\left[\left(n+1\right)!\right]}^{3}}\frac{{\left(ka\right)}^{2(n-1)}}{\left[{\left(n+1\right)}^{2}+\Omega^{2}\right]}$$

Recalling next that under the long wavelength limit ka→0, the helical wave field simply reduces to a *uniformly* rotating field (ROT) with a phasor ${\bar{\chi }}_{am}≃-x_{2}+ix_{3}$, for which case $A_{n}^{1}=(1/2)\delta \left(n-1\right)$. Substituting this value together with Equation (9) in Equation (52) readily renders the well-known ROT expression:

(54) $$T_{DEP}^{\left(1\right)}=-\frac{6\pi \Omega }{4+\Omega^{2}}+O{\left(ka\right)}^{2}$$

previously reported in [13,14,15,17]. Thus, the counter-field ROT/DEP torque has a Lorentzian (‘bell’) shape which vanishes both for zero and infinitely large frequencies with a maximum spectrum amplitude at $\Omega_{\max}=2$. Note that Equation (54) can be also considered as the leading-order long wave approximation of Equation (53), namely keeping only the first term in the summation. Since it has been shown that the ICEP torque for a helical wave forcing is null for a vanishingly small EDL, the resulting dipolophoretic (DIP) torque for a spherical NP consists of only a DEP torque given in Equation (53) in terms of the dimensionless wave number ka.

## 6. Janus Particle

The general expressions obtained so far for the DEP and ICEP forces and torques exerted on spherical particles suspended in a non-uniform (DC or AC) electric fields, are valid both for homogenous metallic NP’s, as well as for MD JP’s. The only difference between the two cases is in the corresponding expressions for the multipoles $D_{n}^{m}$ Equation (3) in terms of the amplitudes $A_{n}^{m}$ of the ambient field, namely Equation (6) for a JP and Equation (9) for a spherical NP.

Let us first consider the DC case of a JP, which is subjected to an arbitrary axisymmetric non—uniform ambient field Equation (1) with m=0. Since for Ω=0 Equation (6) renders $D_{n}=0$, the DC DEP force is given for both JP and NP by (15) as the sum of sequential amplitude terms. Note that this force is null for a uniform field ($A_{n}=0$ for n≠1). Nevertheless, for the general case involving non-uniform ambient fields, the DC ICEP force according to Equation (35) is given by:

(55) FICEP(1)=6πRe{∑n=1nAn[Cn−12n−1−Cn+12n+3]}.

The corresponding expression for the AC ICEP force is given by Equation (35), where the coefficients $D_{n}$ for a JP are found by inverting Equation (6). It is also worth noting that under a uniform ambient field ($A_{n}=0$ for n>1), Equation (55) vanishes for metallic NP’s ($C_{1}\neq 0\;$). Yet, for a JP Equation (55) yields −6π5Re{A1C2} for DC and −3π5Re{A1C2*} for AC excitations respectively, since following Equation (6) both $(C_{n},D_{n})\neq 0$ for any n (even integer under a uniform electric forcing).

Letting, for example, $A_{1}=1$ in Equation (6) and truncating the infinite series after two terms, renders the following set for $\gamma_{1,2}=1/8$ Equation (7):

(56) $$\begin{array}{l} \left(4-i\Omega \right)D_{1}+\frac{3}{8}i\Omega D_{2}=\frac{3}{2}i\Omega \\ \frac{5}{8}i\Omega D_{1}+\left(6-i\Omega \right)D_{2}=-\frac{15}{16}i\Omega \end{array}$$

which yields (4b)

(57) $$D_{1}=\frac{i\Omega C_{1}}{2}=\frac{3\Omega (3i+\frac{49}{128}\Omega^{})}{24-10i\Omega^{2}-\frac{49}{64}\Omega^{2}};\;D_{2}=\frac{i\Omega C_{2}}{3}=-\frac{\frac{15}{4}i\Omega }{24-10i\Omega^{2}-\frac{49}{64}\Omega^{2}}$$

Note that (57b) renders $C_{2}=-15/32$ for Ω=0. Finally, substituting this value into Equation (55) and recalling (charge conservation) that $C_{0}=0$, simply yields $F_{ICEP}^{\left(1\right)}=-\frac{9\pi }{16}$ or (dividing by 6π) $U_{ICEP}=-\frac{3}{32}$, in agreement with Equation (3.16) of Squires & Bazant [8]. The resulting JP ICEP velocity is directed along the $x_{1}$ axis, namely from the metallic toward the dielectric hemisphere. Thus, it is shown that due to symmetry- breaking, a JP (unlike common NP) will experience a finite ICEP force/velocity even under a uniform DC electric excitation.

A similar procedure can be readily applied to evaluate the corresponding forces acting on a JP which is subject to a uniform DC or AC ambient field applied in the transverse directions ($x_{2},x_{3}$). This case can be envisaged by letting m=1 in Equation (1) and considering for example $A_{n}^{1}=\delta \left(n-1\right)$. Since according to Equation (9), $\left(C_{n}^{1},D_{n}^{1}\right)=0$ for n≠1, the ICEP force Equation (35) for a metallic NP is again null as expected. Nevertheless, the corresponding ICEP force Equation (44) for a JP is generally non-zero (even for a uniform ambient field) and is given by

(58) FICEP(1)=3πRe{∑n=1n(n+2)[n+12n+1An+11C*n1−n2n+3An1C*n+11]}

which for $A_{1}^{1}=1$ simply yields FICEP(1)=−(9π/5)Re{A11C2*1}. Recalling next that $\gamma_{1,2}^{1}=3/4$ Equation (7), substituting this value into Equation (6) and using again only a two-term expansion, yields for $\left(D_{1},D_{2}\right)$:

(59) $$\begin{array}{l} \left(4-i\Omega \right)D_{1}^{1}+\frac{9}{8}i\Omega D_{2}^{1}=\frac{3}{2}i\Omega \\ \frac{5}{8}i\Omega D_{1}^{1}+\left(6-i\Omega \right)D_{2}^{1}=-\frac{15}{16}i\Omega \end{array}$$

Solving the above set for Ω=0 renders $C_{2}^{1}=D_{2}^{1}/\left(3i\Omega \right)\to -\frac{15}{32}$. Thus, the corresponding ICEP force is $-\frac{27\pi }{32}$ or $U_{ICEP}^{\left(1\right)}=-\frac{9}{64}$, which again coincides with Equations (3.16) of [8]. Finally, we note that such a transverse forcing (parallel to JP interface), will also induce an ICEP velocity whereby the JP tends to move in the axial direction towards its dielectric part.

As for a JP that is subjected to an arbitrary (non-uniform) AC field, the preceding analysis holds as well, providing the coefficients $D_{n}^{m}$ are determined by inverting Equation (6) in terms of the prescribed forcing amplitudes $A_{n}^{m}$. The expressions obtained in the previous sections for travelling-waves (i.e., linear m=0 or circumferential m=1), may be also applied for a JP. It is worth mentioning that the common case of a *uniform* field (axial or transverse), either under DC or AC forcing, can be directly obtained from the solutions found for linear or helical travelling waves under the limit of the ‘long-wave’ approximation, i.e., ka→0. 

We demonstrate below how the familiar ROT spectra for a freely suspended spherical NP, can be easily extended for the case of a MD JP. For this purpose, we make use of the general expression found for the DEP torque Equation (52), by noting that for a metallic NP it renders Equation (54), where $D_{1}^{1}=\frac{3}{2}i\Omega /\left(2-i\Omega \right)$. The same expression Equation (52) still holds for a JP providing the $D_{n}^{1}$ coefficients are found from Equation (6) or Equation (59). In particular, if we approximate $D_{1}^{1}$ by solving Equation (59) (i.e., including only the two leading terms), then Equation (52) simply yields for n=1 and $A_{1}=1$:

(60) TDEP(1)=−6πIm{3iΩ+19128Ω224−10iΩ−1964Ω2}.

On the other hand, if we consider only ‘one-term ‘ approximation in Equation (59), one gets $D_{1}^{1}=\frac{3}{2}i\Omega /\left(4-i\Omega \right)$ and the corresponding JP torque is again of a Lorentzian type, given by $T_{DEP}^{\left(1\right)}=-12\pi \Omega /(16+\Omega^{2})$. These two approximate (i.e., ‘one’ and ‘two” term) solutions for a spherical JP, can be compared against the prevalent ROT solution Equation (54) for a spherical (metallic) NP.

Finally, it is important to note that unlike perfectly conducting spherical colloids, for which the ROT spectrum is known Equation (54), the corresponding spectrum for a metallo-dielectric Janus particle has not been obtained. The common practice to estimate ROT spectra for MD JP’s, is by taking the average between the effective CM (Clausius—Mossotti) coefficients of the dielectric and metallic phases [52]. This procedure, when applied to a MD JP, reduces the spectrum amplitude approximately by a factor of 2 (since the dielectric permittivity is ignored with respect to the metallic), but still its peak remains at $\Omega_{\max}^{\left(NP\right)}=2$. Measurements of ROT spectrum [52], for a Pt-silica JP (normalized with respect to the volume of the metallic phase) in DI, indicates that the JP spectrum is indeed shifted (compared to NP) to higher frequencies (see Figure 4a in [52]), in accordance with the above simplified ‘one-term’ approximation resulting in $\Omega_{\max}^{\left(JP\right)}=4$.

## 7. Levitation: JP in a Pore

In order to demonstrate the preceding methodology for a Janus particle, let us consider the levitation problem of a MD JP of radius *a* that is freely suspended in a solute within a vertical cylindrical pore of height *H* and bounded by two circular electrodes (Figure 3). If both electrodes are grounded, the heavy JP will rest due to gravity on the bottom of the container with its metallic hemisphere facing downwards. We use a cylindrical coordinate system (x,r) attached to the lower electrode (LE) at x=0, which is assumed to be powered to a potential $-V_{0}J_{0}\left(\lambda r\right)$, where the upper electrode (x=H) remains grounded. Here $V_{0}$ represents the maximum potential at the center (r=0) of LE (b≥r≥0) and λ is an arbitrary parameter. The electric potential (Laplace) induced in the cylindrical domain is clearly given by $-V_{0}\frac{\sinh[\lambda \left(H-x\right)]}{\sinh\left(\lambda H\right)}J_{0}\left(\lambda r\right)$. The reader is reminded that λ=38317/b if the cylindrical (r=b) walls are insulating ($J_{1}\left(\lambda b\right)=0$) and λ=2.408/b if the walls are conducting ($J_{0}\left(\lambda b\right)=0$). The polarized JP will then experience both DEP and ICEP axial forces which will either push (levitate) the JP away from the LE or pull it toward the bottom depending on the ambient forcing. We will next discuss the levitation dynamics of a JP under such non-uniform excitations.

Assuming that under the combined DIP (DEP+ICEP) forces, both NP and JP, are levitated (positive DEP) to a distance x=c (Figure 3) above the LE. Following Section 3.2.2 and when expressed in a body-attached coordinate system ($x_{1},r$) such that $x_{1}=x-c$, the ambient potential is given by Equation (19) (multiplied by $V_{0}$). Let us first consider a spherical NP, where the DEP (pointing upward) force is given explicitly by Equation (21). The corresponding ICEP and DIP expressions, are given by Equations (37) and (38) respectively in terms of the prescribed amplitudes Equation (20). Substituting Equation (20) into Equation (38), renders the following dimensional expression for the vertical DIP force acting on a NP under such exponentially decaying field:

(61) $$F_{DIP\left(NP\right)}^{\left(1\right)}=2\pi \varepsilon V_{0}^{2}\frac{\sinh\left[2\lambda \left(H-c\right)\right]}{\sinh^{2}\left(\lambda H\right)}\Theta \left(\lambda a\right);\;\;\Theta \left(z\right)=zI_{2}\left(2z\right)-\frac{3I_{2}\left(2z\right)}{z}+\frac{3z}{2}$$

where ε is the solute dielectric constant $I_{2}(z)$ and denotes the Bessel function. Note that Equation (61) is *exact* and that the DIP force in this case Equation (19) is pointing upward (opposite to gravity) since Θ is positively definite. As a result, a metallic NP of effective mass $m_{eff}$ (including buoyancy) will be always lifted (levitated) from the bottom to an equilibrium distance $\bar{c}$ from the LE. Assuming next that H/b>>1 (or λH>>1) as well as $2\pi \varepsilon V_{0}^{2}\Theta \left(\lambda a\right)>m_{eff}=(4/3)\pi a^{3}g(\rho_{M}-\rho_{F})$ and recalling that under static equilibrium $F_{DIP}^{\left(1\right)}=m_{eff}g$ results in:

(62) $${\bar{c}}_{NP}=\frac{1}{2\lambda }\ln\left(\frac{3\varepsilon V_{0}^{2}\Theta \left(\lambda a\right)}{{\tilde{\rho }}_{NP}ga^{3}}\right);\;\;{\tilde{\rho }}_{NP}=\rho_{M}-\rho_{F}$$

Here, $\rho_{M}$ denotes the density of the metallic NP, $\rho_{F}$ represents the density of the fluid and g is the gravity acceleration. Further simplifications of Equation (62) are possible for λa<<1 (or b≫a), by noting following Equation (61b) that $\underset{z\to 0}{\lim\;\;\;}\Theta \left(z\right)=\frac{5}{16}z^{5}+O\left(z^{7}\right)$ where the term in the parenthesis in Equation (62) can be replaced by $15\lambda^{5}\varepsilon V_{0}^{2}a^{2}/16{\tilde{\rho }}_{NP}g.$

It is important to note that this levitation equilibrium point is *stable* to disturbances in the radial direction, since the maximum potential (positive CM coefficient), is located on the axis (r=0). Thus, any small radial displacement of the NP from the axis will result in a finite restoring DEP force directed toward the axis (similar to an optical tweezer [53]). As far as the stability in the axial direction is concerned, we recall following Equation (21) that $F_{DIP}^{\left(1\right)}\left(x_{1}\right)=F_{DIP}^{\left(1\right)}\left(0\right)e^{-2\lambda x_{1}}$, where $x_{1}$ denotes a small axial displacement from the equilibrium point, i.e., $\bar{x}=\bar{c}+x_{1}$ and $F_{DIP}^{\left(1\right)}\left(0\right)$ is given in Equation (61a). Linearizing the dynamic equation, which governs the axial motion of a metallic NP around the equilibrium point ($x_{1}=0$), finally yields [4,40]:

(63) $$\frac{4\pi }{3}g\rho_{M}a^{3}\left(1+q\right){\ddot{x}}_{1}+6\pi \eta a{\dot{x}}_{1}+2\lambda F_{DIP}^{\left(1\right)}\left(0\right)x_{1}=0$$

where $q=\rho_{F}/(2\rho_{M})$ (added—mass correction) and η denotes the dynamic viscosity of the ambient fluid. The first term in Equation (63) represents NP inertia and the second is the (Stokes) damping force (ignoring wall effects). Since both $F_{DIP}^{\left(1\right)}\left(0\right)$ and λ are positive, stability in the axial direction is also assured. Thus, the NP exhibits a *passive* stable levitation behavior [40,41], by executing small damped oscillations about the equilibrium point $x=\bar{c}$.

The corresponding levitation problem for a JP (in contrast to a NP) under the same electric excitation, is somewhat more intricate since the expression for the DIP force has to be modified in order to account for symmetry- breaking effects related to the large disparity between the dielectric constants of the two hemispheres comprising the MD JP. The DEP force acting on a JP, is still given by Equation (21) (exact), namely $F_{DEP}^{\left(1\right)}∼2\pi {\left(\lambda a\right)}^{3}e^{-2\lambda \bar{c}}+O{\left(\lambda a\right)}^{5}$ for λa→0 and λH>>1. Nevertheless, the JP ICEP force, can be found from Equation (35) by noting that the multipole coefficients $D_{n}$ are given by Equation (6) in terms of the ambient amplitudes $A_{n}$. In particular, using Equation (4b) together with Equation (6) implies for example that in the DC limit (Ω=0), one gets the following explicit expression:

(64) $$\frac{2}{2n+1}C_{n}=\frac{A_{n}}{n+1}-\sum_{l\neq n}\frac{2l+1}{l+1}\gamma_{e,n}A_{l}$$

and thus following Equation (35);

(65) FICEP(1)=6πRe{∑n=1(n+12n+1CnAn+1−n2n+3Cn+1An)}.

Recalling next that for λH>>1, Equation (20) implies that $A_{n}=\frac{{\left(-1\right)}^{n+1}}{n!}{\left(\lambda a\right)}^{n}e^{-\lambda \bar{c}}$ and thus the coefficients $A_{n}$ are asymptotically small for λa→0. Therefore, the leading- order ICEP force for a JP, can be written following Equations (64) and (65) $\left(\gamma_{1,2}=1/8\right)$ simply as $6\pi A_{1}[\frac{3}{32}A_{1}+\frac{1}{3}A_{2}]+{\left(\lambda a\right)}^{4}$. Finally, combining ICEP with the leading ‘two- term’ DEP expression Equation (15), namely $-4\pi A_{1}A_{2}$ Equation (15), we obtain the corresponding DIP expression for a JP (assuming λa≪1):

(66) $$F_{DIP\left(JP\right)}^{\left(1\right)}=6\pi A_{1}\left[\frac{3}{32}A_{1}-\frac{1}{3}A_{2}\right]+O{\left(\lambda a\right)}^{4}=3\pi {\left(\lambda a\right)}^{2}e^{-2\lambda \bar{c}}\left[\frac{3}{16}+\frac{\lambda a}{3}\right]+O{\left(\lambda a\right)}^{4}.$$

Consider for example the case of an ambient DC electric field with a ‘constant gradient’ [8,9,17], namely where only $A_{1}$ and $A_{2}$ are nonzero. We recall following Equation (38) that the DIP force acting on a NP is null regardless of the values of these two amplitudes, whereas the corresponding DIP force for a JP Equation (66) is finite. It is also worth mentioning that according to Equation (66) the JP DIP force vanishes to $O{\left(\lambda a\right)}^{4}$ providing $A_{2}=\frac{9}{32}A_{1}.$

As far as the corresponding levitation problem of a JP is concerned, one can repeat the analysis leading to Equation (62) and show that a JP of same radius and forcing as that of a NP, is levitated instead to an equilibrium height ${\bar{c}}_{JP}>>{\bar{c}}_{NP}$, where

(67) $${\bar{c}}_{JP}=\frac{1}{2}\ln\left(\frac{27\varepsilon V_{0}^{2}{\left(\lambda a\right)}^{2}}{32{\tilde{\rho }}_{JP}ga^{3}}\right);\;\;\;2{\tilde{\rho }}_{JP}=\rho_{M}+\rho_{D}-\rho_{F}$$

Here, $\rho_{D}$ denotes the density of the dielectric and $\rho_{M}$ the metallic parts of the JP hemispheres respectively. Finally, comparing Equations (62) and (67) we find that (λa≪1) levitation effects are more pronounced (enhanced) for a JP compared to a NP (∼ by a factor of 5/2). It is also worth mentioning that dividing Equation (66) by the Stokes drag coefficient 6π, indicates that when exposed to a uniform ambient field (i.e., $A_{n}=0$ for n≠1), a MD JP acquires a dimensionless ICEP velocity (directed towards its dielectric part) given by 3/32 in agreement with Equation 3.16 of [8].

## 8. Summary and Discussions

In this study we present a general framework for calculating the DEP and ICEP dynamic loads (forces and moments) acting on initially uncharged perfectly conducting (metallic) spherical nano/micro and Janus particles exposed to an arbitrary (DC or AC) non-uniform ambient electric fields. The analysis is carried under the assumption of ‘weak’ field (ignoring convection & surface conductance) and infinitely small Debye scale. The above procedure enables us to solve the coupled linearized PNP system and obtain a closed form solution for both electrostatic and hydrodynamic problems. Special attention is payed to metallo-dielectric JP and to inhomogeneous travelling -wave type electric forcing with a prescribed wavelength and frequency. Analytic expressions can be thus found for twDEP and twICEP, which allows us to check the accuracy of the available approximations against the exact value for different wavelengths.

The imposed non-uniform ambient field is expanded in general spherical harmonics in terms of prescribed (complex) amplitudes $A_{n}^{m}$, where n and m≤2n+1 are positive integers, denoting the order and mode of the harmonic forcing respectively. Expressed in a Cartesian coordinate system $\left(x_{1},x_{2},x_{3}\right)$ such that $x_{1}$ denotes the axis of symmetry, implies that m=0 corresponds to the case of an axisymmetric loading and n represents the order of the polynomial in the Cartesian coordinates. It is first demonstrated that under general inhomogeneous electric forcing, the NP experiences DEP and ICEP axial loadings (in $x_{1}$ direction) due to Re{AnmAn+1m} type interactions (namely between odd and even amplitudes of same order). In a similar manner, transverse DEP and ICEP forces (along $x_{2}$ or $x_{3}$ directions), arise from Re{AnmAn+1m±1} type interactions. On the other hand, a spherical NP is subjected to a torque acting in the axial direction as a result of Im{AnmAn+1m} type interaction and a similar one in the transverse direction, where Re and Im denote the real and imaginary parts respectively. Corresponding analytic expressions for the dynamic reactions on a MD JP (exhibiting material symmetry breaking) are also provided in terms of the coefficients $D_{n}^{m}$, which are related to $A_{n}^{m}$ through Equation (6). Using the above formulation, one can demonstrate that under the ‘constant gradient’ or ‘linear’ approximation, where only the first two terms corresponding to n=1 and n=2 are considered, the DEP and ICEP (at least for low frequencies) act in opposite directions. Moreover, in the DC limit, it is verified that the DIP (sum of DEP and ICEP), indeed vanishes for spherical NP. However, as shown this interesting property does not hold under AC forcing (and even in DC for finite EDL’s). 

In order to demonstrate the above methodology, we consider in particular electric forcing of a freely suspended NP by both standing and travelling waves in a cylindrical container. Exact expression is found for the DEP force exerted on a NP placed a standing wave Equation (16) in terms of its wavelength, size of NP and a parameter c (Figure 2) representing its position with respect to the nearest wave nodal point Equation (18). The DEP vanishes if c is null. A similar analytic form Equation (21) is found for an exponentially decaying (converging) field (Figure 3) due to powered and grounded circular electrodes Equation (19). These axisymmetric solutions are then extended for helical wave excitations Equation (27) again resulting in rather simple exact expressions Equation (29). A similar procedure can be used to calculate ICEP for travelling waves in terms of the characteristic (RC) frequency. For example, closed form solution can be obtained for both DEP and ICEP forces acting on a perfectly polarized spherical NP subject to a general travelling wave of wavelength k and frequency ω. Such solutions are given in Equations (30) and (41) in terms of the dimensionless wavenumber ka and frequency Ω and thus can be finally compared against the available approximate solutions.

**Figure 4:**
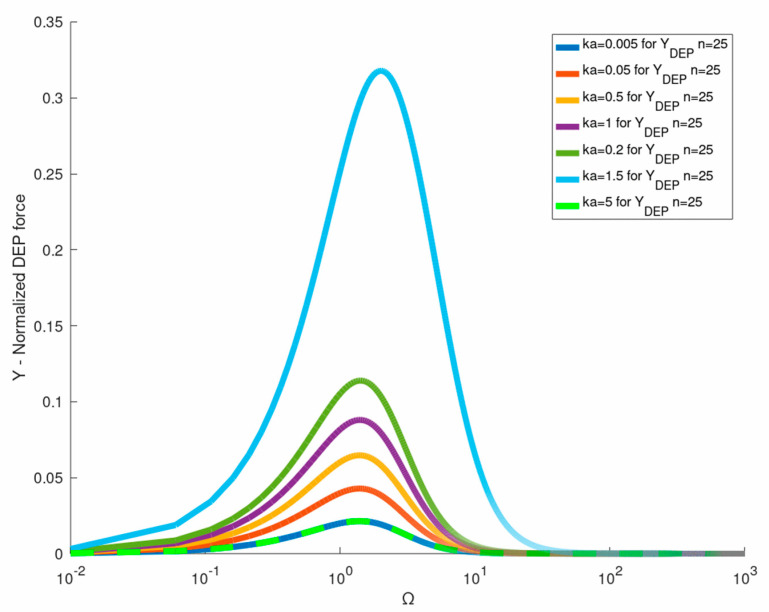
Comparison of the exact expression for the twDEP force (Equation (41)) for different ka values against the ‘long wave’ approximation (n=1).

**Figure 5:**
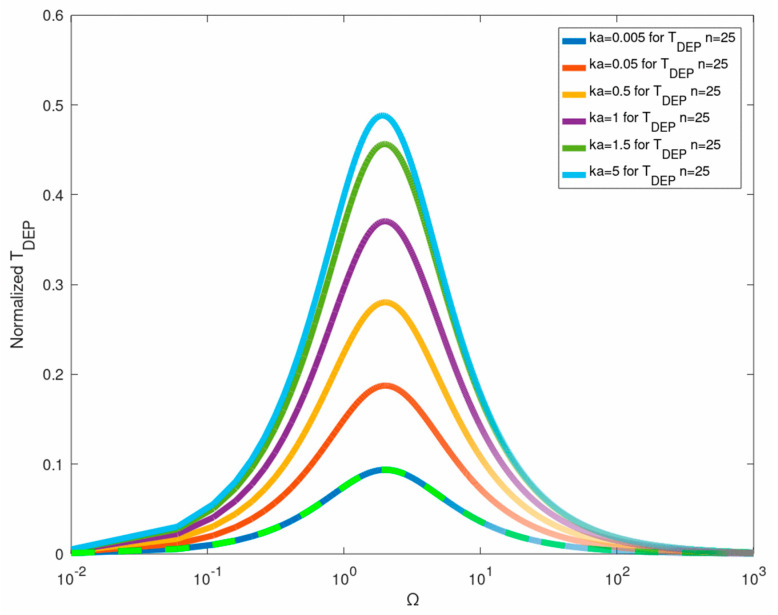
A comparison between the exact (Equation (53)) twDEP torque spectrum (normalized by 16π) for various ka values, against the corresponding ‘long-wave’ approximation (Equation (54)).

Figure 4 is a plot of the exact ICEP expression Equation (41) versus ka and Ω against the ‘constant-gradient’ (n=1) approximation recently provided by Equation (27) in [27]. It shows that the two-term ‘constant-gradient’ approximation can be used only for extremely small dimensionless wavelength ka of the order of $10^{-2}$! Similar exact expressions can be obtained for the DEP torque acting on a NP under a helical (circumferential) travelling wave Equation (27) (note that the ICEP torque vanishes in this case). The exact solution for the DEP torque Equation (53) is compared in Figure 5 against the common leading- order Equation (54) ROT solution [13,17]. Again, one finds that Equation (54) can be considered as the small wavelength (ka→0) limit (Rayleigh) of the exact solution which can be used for ka≤0.05. The DEP force (Figure 4) and torque (Figure 5) spectra are of Lorentzian (bell) shape, vanishing for small and large frequencies with a distinct maximum at Ω∼2. The same approach can also be used to evaluate the gradient force (optical tweezer) exerted on a NP which is subjected to a non-uniform BB (Bessel beam) under the Rayleigh approximation.

Unlike homogeneous NP, spherical JP’s generally experience ICEP even under *uniform* ambient fields Equation (58). In particular we find that for Ω=0 (DC limit), the dimensionless ICEP velocities in the direction normal to the interface (toward the dielectric hemisphere) are given by 9/64 and 3/32 for a field directed parallel or normal to JP interface respectively, in full agreement with [8]. An analytical expression for the ICEP loading is also provided Equation (58) for arbitrary AC and non-uniform electric forcing in terms of the complex amplitudes of the ambient field. The DIP dynamic loads acting on both NP and JP exposed to a non- homogeneous ambient field, depend on the interactions between the ambient amplitudes $A_{n}^{m}$ and the multipole term $D_{n}^{m}$ Equation (3). Note that for a metallic NP, the coefficients $D_{n}^{m}$ are given explicitly in terms of $A_{n}^{m}$ Equation (9a), whereas for JP they are found by solving a linear system Equation (6).

**Figure 6:**
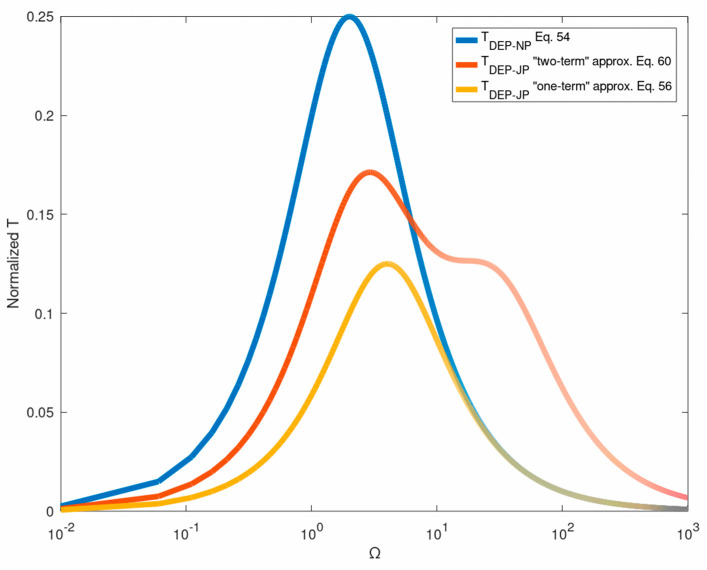
Comparison between the ‘two-term’ (Equation (60)) approximate ROT spectra for the JP torque against the corresponding spectrum for a NP (Equation (54)). Also shown is the ‘one-term’ JP approximation.

The case of a JP, which is subjected to ROT ambient field consisting of two orthogonal out-of- phase components parallel to the JP interface, is also of special interest. Letting for example $A_{1}^{1}=1$, Equation (52) implies that the DEP torque depends on the imaginary part of the coefficient ${\tilde{D}}_{1}^{1}$ Equation (35b), found by inverting the linear system Equation (6). A one-term solution of Equation (6) is $D_{1}^{1}=(3i/2)/(4-i\Omega )$ resulting Equation (54) in $T_{DEP}^{\left(1\right)}=-12\pi \Omega /(16+\Omega^{2})$. Equation (57a) represents the corresponding ‘two-term’ solution of Equation (6) leading to the JP torque spectrum given by Equation (60). Comparing these two approximate spectra in Figure 6 against the equivalent one for a NP Equation (54), indicates that the JP spectra are generally lower (depending on coating thickness) and are shifted towards higher frequencies in qualitative agreement with the available measurements [53].

The weak ‘double-peak’ frequency response curve depicted in Figure 6, may be a unique feature of JP (compared to the Lorentzian-type NP dispersion). Nevertheless, recall that the above JP spectrum Equation (60) has been obtained as a ‘two-term’ approximation of Equation (6), whereas the corresponding ‘one-term’ approximation is again of a ‘bell’ shape. Obtaining more accurate JP frequency spectra are underway. It is also important to note that so far, no attempt has been made to analytically calculate the JP spectra for AC excitation and the common practice is to express it in terms of the average between the CM coefficients of the metallic and dielectric phases. Such an approximation clearly fails to predict the reported physical JP frequency ROT shift with respect to a NP [53] as depicted in Figure 6. The theoretical model also indicates that the characteristic frequency $\Omega_{\max}$ Equation (4c) is inversely proportional to the JP radius and EDL thickness (through the $x^{-1/2}$ dependence on solute conductivity), in agreement with experimental findings.

Levitation and stability issues of both NPs and JPs under general non-uniform electric excitations and in particular under travelling wave and converging (decaying) fields have been also discussed. It is demonstrated that freely suspended spherical polarizable NP’s and JP’s placed near the lower powered electrode along the axis of a cylindrical (insulating or conducting walls) pore, are levitated due to positive DEP (overcoming gravity) to an equilibrium distance $\bar{c}$ from the bottom. As shown, this equilibrium point is stable with respect to both radial and radial disturbances. It is also interesting to note that JP’s, as compared to common NP’s, are more amenable to DEP levitation (by a factor 2–3), exhibiting yet another remarkable property of asymmetric Janus particles in response to ambient non-uniform electric fields.
